# Farinacea in the Diet of Later Infancy

**Published:** 1898-09

**Authors:** 


### Farinacea in the Diet of Later Infancy.

Miller writes on this subject in the *Archives of Pediatrics* (April). He says it is an every day experience to see an infant who has thriven upon the breast or artificial food during its first year, suffer continually from digestive disturbances when that period has passed, and this is largely due to adding to the dietary, under the impression that the former dietary is no longer nourishing enough, pure milk, bread, potatoes, crackers and strong porridges. This persisted in leads to chronic intestinal indigestion. The child during the second year does not need the large proportion of carbohydrates it receives in earlier life, *i. e.*, 6 of carbohydrates to 1 of proteid, and is not able to assimilate them if given in large amounts in farinaceous food. The child at this period requires more nitrogen in proportion to other food elements than in the first year, and to secure its complete oxidation this should be given with the minimum of carbohydrates. Potatoes should not be given until the twenty-fourth month and then always baked. Orange juice supplies their antiscorbutic properties very well. Potatoes and oatmeal are the most active causes of indigestion at this age. All farinacea should occupy a secondary place in the child's diet until the child has shown his capacity to digest them, which is usually not before the twenty-fourth month.

